# Autistic people differ from non-autistic people subjectively, but not objectively in their reasoning

**DOI:** 10.1177/13623613241277055

**Published:** 2024-10-10

**Authors:** Elif Bastan, Sarah R Beck, Andrew DR Surtees

**Affiliations:** 1 University of Birmingham, UK; 2 Karadeniz Technical University, Turkiye; 3 Birmingham Women’s and Children’s NHS Trust, UK

**Keywords:** autism, deliberation, dual process theory, intuition, reasoning

## Abstract

Autism has been linked to difficulties within the social domain and quick decision-making. The Dual Process Theory of Autism proposes that autistic people, compared to non-autistic people, tend to prefer and perform in a more deliberative and less intuitive reasoning style, suggesting enhanced rationality in autism. However, this theory has not been systematically explored across social and non-social domains. In total, 24 autistic adults and 24 age-, gender- and non-verbal cognitive ability–matched non-autistic counterparts completed subjective and objective measures of reasoning. A scenario-based comparison task was employed, covering both social and non-social domains, to assess whether participants consistently used the same strategy across domains or alternated between strategies for their forced-choice judgements. On a subjective measure of reasoning, compared to their counterparts, autistic participants self-reported lower intuition. However, there was an opposite pattern on an objective measure, in that autistic participants responded more intuitively. No significant group differences were identified between autistic and non-autistic participants on self-reported deliberation, objectively measured reflectiveness or forced-choice moral judgements across social and non-social domains. There might be a discrepancy among autistic people for their subjective preferences and objective performances in reasoning, decision-making and judgements.

**Lay Abstract:**

Autistic people often experience challenges in social contexts, and when decisions need to be made quickly. There is evidence showing that autistic people have a tendency for greater deliberation and lower intuition, compared to non-autistic people. This has led to the researchers’ proposal that autism is associated with an enhanced level of rationality. However, these theories have been mostly explored through the lens of either only non-social domain or only social domain. To address this gap, we recruited autistic adults and carefully matched them with non-autistic adults for comparison. We used a task representing both social and non-social interactions in a comparison structure and asked participants’ moral judgements on scenarios’ main characters. This was complemented by subjective and objective measures of reasoning. Our findings did not reveal meaningful differences between groups in terms of deliberation. However, we did observe that autistic participants self-reported lower levels of intuition, compared to non-autistic participants. Autistic people consistently rate themselves as less intuitive than their counterparts. Nevertheless, objective evidence supporting this across tasks and studies is inconsistent.

Autism is characterised by differences from non-autistic people in social communication and interaction, alongside repetitive, stereotyped and restricted behaviours, activities and interests (American Psychiatric Association, 2013). One specific area of social cognition in which autistic people^1^ differ from non-autistic people is their *preference* and *performance* in reasoning and decision-making (Morsanyi & Byrne, 2020; Rozenkrantz et al., 2021; van der Plas et al., 2023). Reasoning is crucial for decision-making, affecting various aspects of life, including independence and employment.

## Dual process theory of autism

Dual Process Theories have recently been employed to explore how autistic people reason and make decisions. Dual Process Theories, widely used in cognitive and behavioural studies to investigate reasoning, propose two information processing types: fast ‘intuition’ and slow ‘deliberation’. While there is an ongoing debate about this distinction (De Neys, 2018), intuition refers to a quick, effortless and automatic process, mainly used for spontaneous and instant judgements. However, deliberation refers to a slower, more effortful and less automatic process, mainly used for complicated and remarkable decisions (Evans, 2008, 2011; Kahneman, 2011). According to *the default-interventionist model*, intuition serves as the default mode for everyday reasoning, unless decisions are re-evaluated through deliberation (Evans & Stanovich, 2013).

Dual Process Theory of autism proposes that autistic people, compared to non-autistic people, demonstrate *preferences* and *performance* in greater deliberation and reduced intuition, alongside less engagement in common cognitive biases, suggesting enhanced rationality in autism (e.g. Ashwin & Brosnan, 2019; Brosnan & Ashwin, 2023a, 2023b; Brosnan et al., 2016, 2017; De Martino et al., 2008; Levin et al., 2021; Lewton et al., 2019; Rozenkrantz et al., 2021). This theory aligns with other characteristics linked to autism, such as enhanced *attention-to-detail*, where autistic people tend to focus on details before considering concepts, *the bigger picture*, leading to longer processing times (Baron-Cohen et al., 2009). Together with *hyper-attention*, *hyper-systemising* skills, which involve the capacity to identify and manipulate causal patterns, have been suggested to stimulate autistic strengths (Baron-Cohen, 2020). In contrast, non-autistic people tend to favour *top-down thinking*, where they consider concepts, before delving into details. Ostensibly, autistic people’s approach demands more time and effort, yet it reduces the risk of overlooking important information. However, greater deliberation and reduced intuition in autism might have potential downsides. Because autistic people reportedly tend to rely less on fast intuition compared to non-autistic people who regularly engage in this kind of information processing (Rand, 2016), autistic people may face challenges in rapidly changing social situations that often necessitate quick decision-making based on subtle social cues. For instance, in scenarios such as job interviews, longer response times might negatively influence an interviewer’s perception of the candidate.

## Evidence for greater deliberation in autistic people

A *preference* and *engagement* in greater deliberation in autistic people has been highlighted in several studies (e.g. Ashwin & Brosnan, 2019; Brosnan & Ashwin, 2023b; Brosnan et al., 2016, 2017; De Martino et al., 2008; Levin et al., 2021; Lewton et al., 2019; Rozenkrantz et al., 2021). Studies using subjective self-report measures, such as the Rational Experiential Inventory (REI; Epstein et al., 1996) and objective performance-based measures, such as the Cognitive Reflection Test (CRT; Frederick, 2005), reported that autistic people tend to score higher in deliberation and lower in intuition. In the classical CRT (Frederick, 2005), each question has a correct and an incorrect response. In this test, correct responses indicate deliberation, and incorrect responses indicate intuition. The CRT evaluates one’s capacity to resist incorrect ‘gut’ feelings and instead engage in deeper reflection to arrive at the correct answers. Consisting of three questions, each with a seemingly obvious yet incorrect response, the classical CRT serves as a tool to gauge this cognitive tendency. Brosnan et al. (2016) employed the classical CRT to compare reasoning performance between autistic and non-autistic people. They reported that autistic people provided more correct and fewer incorrect responses compared to non-autistic people. In addition, autistic people’s self-reports using the REI, highlighted higher rationality scores, indicative of deliberation, and lower experientiality scores, indicative of intuition. Brosnan et al. (2016) concluded that autistic people are not ‘lazy thinkers’ as they adopt a reasoning strategy that requires more time and effort yet leads to greater accuracy.

Autistic people also show evidence for reduced engagement in common cognitive biases (De Martino et al., 2008; Farmer et al., 2017; Fujino et al., 2020; Shah et al., 2016; Vella et al., 2018). For instance, autistic people typically do not *jump to conclusions* (Brosnan et al., 2014), a bias that occurs when a decision is made prematurely with insufficient information. Brosnan et al. (2014) used the Beads Task (Huq et al., 1988) to compare autistic adolescents to an age-matched non-autistic group. The Beads Task involves two jars filled with different coloured beads with different distributions. Participants draw beads from each jar. Participants must determine which of the two jars beads are being drawn from. The study found that autistic participants requested more beads before reaching a decision compared to non-autistic participants who made decisions with fewer beads. This suggests that autistic people adopt a slower yet more careful reasoning approach. Similar results were observed with the general population, where participants with higher autistic traits requested more information prior to decision-making compared to those with lower autistic traits (Brosnan et al., 2013). These findings are consistent with self-reports from autistic people and their caregivers indicating challenges of quick decision-making (Luke et al., 2011).

## Limitations of the literature

There are discrepancies in the literature on reasoning and decision-making in relation to autism. Reasoning performance is closely linked to cognitive abilities; therefore, adjusting for cognitive ability might impact the results. For instance, the link between autism and greater deliberation was not found when comparison groups were matched on cognitive ability or when cognitive ability was adjusted (e.g. Brosnan et al., 2017; Jänsch & Hare, 2014; Morsanyi & Hamilton, 2023; Taylor et al., 2022). Based on four large-scale studies, Taylor et al. (2022) did not report associations between autism and objectively measured deliberative and intuitive reasoning. Even when they did identify significant associations, adjusting for cognitive ability between groups rendered these associations insignificant. The single link they found was between autism and self-reported greater intuition. Moreover, many studies relied on the classical CRT, which has been widely spread through newspapers and online platforms, while neglecting to measure the familiarity with its items (e.g. Brosnan & Ashwin, 2023b; Brosnan et al., 2016, 2017).

Jänsch and Hare (2014) also employed the Beads Task, after carefully matching autistic adults to a comparison group based on age, gender and cognitive ability. Contrary to Brosnan et al.’s (2014) findings, they found that to reach a decision autistic adults required fewer beads compared to the comparison group. Autistic adults made quick decisions based on only one bead drawn from a jar in half of the trials, while none non-autistic adult displayed such pattern. These two studies, published around the same time, present conflicting results. The Beads Task is also subject to criticism based on low ecological validity (e.g. Westermann et al., 2012). In addition, several studies recruited non-clinical samples by measuring autistic traits (e.g. Lewton et al., 2019). While this strategy allows recruitment of larger samples, results cannot be generalised to the autistic community (Sasson & Bottema-Beutel, 2021).

## Moral reasoning in autism

**Figure 1.**
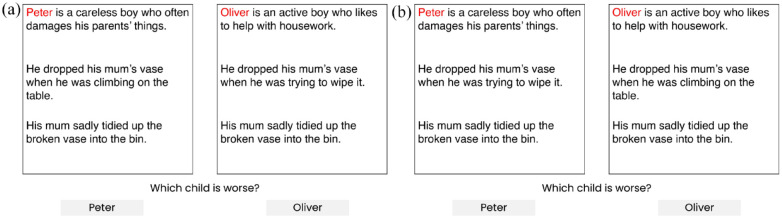
Example (a) consistent and (b) inconsistent scenario comparisons in the social domain. Source: Adapted from Komeda et al.’s (2016) work. For comparisons with good outcomes, participants were asked which child was better, and for comparisons with bad outcomes, which child was worse.

Moral reasoning is reasoning about what is good or bad, right or wrong, permissible or impermissible, prescribed or proscribed, *inter alia*. An example in the literature brings light to differences in moral reasoning across autistic and non-autistic young people. Komeda et al. (2016) showed participants three-line vignettes describing interactions between a child and the child’s parent (see Figure 1). The first line of each scenario described the child’s character, the second line depicted the child’s behaviour and the third line provided the outcome of the scenario. Each line had either a positive or negative valence, and the scenario structures varied in consistency. Participants were asked to judge which child was better or worse. Komeda et al. (2016) found that autistic young people more consistently relied on behaviour-based information in making these judgements, whereas their non-autistic counterparts tended to use both character- and behaviour-based information. This suggests autistic young people to engage in more rational patterns of reasoning, whereby they consistently relied on the same kind of information for each scenario while ignoring the characteristics of the social agents (also see Moran et al. (2011) which suggests difficulties among autistic participants in integrating mental state information of social agents for moral judgements).

## Social versus non-social reasoning

To draw conclusions on social cognition, it is important to examine social phenomena in comparison with a non-social context (Lockwood et al., 2020). Performance across social and non-social realms may differ because deploying cognitive information is likely to be influenced by context. Autistic people reportedly exhibit enhanced reasoning abilities within non-social realm. For instance, Scott and Baron-Cohen (1996) suggested that autistic people face challenges in social–psychological reasoning, while they show enhanced abilities in non-social logical reasoning. While social and non-social reasoning have been studied in relation to autism mostly in isolation of a single domain, a systematic comparison across these domains has been overlooked.

## This study

In this study, we adapted Komeda et al.’s (2016) social scenarios and created structurally equivalent non-social scenarios to examine reasoning differences between autistic and non-autistic people across social and non-social domains. We also used the REI, as a subjective measure of reasoning, and an updated version of the CRT, as an objective measure of reasoning. Following the prevailing literature on the Dual Process Theory of autism, we hypothesised that the autism group would (1) self-report higher levels of rationality, indicative of deliberation, and lower levels of experientiality, indicative of intuition, compared to the non-autistic group on the REI, (2) outperform the comparison group by providing more correct/deliberative and less incorrect/intuitive responses on the CRT and (3) demonstrate more consistent reasoning by providing a greater proportion of behaviour-based responses for social scenario comparisons, compared to the non-autism group, while no significant group differences were expected for non-social scenario comparisons.

## Methods

For this study, the hypotheses and analysis plan were specified prior to data collection. See https://osf.io/vjb7x/ for pre-registration, data and material. This project was conducted following the British Psychological Society ethical guidelines and approved by the Science, Technology, Engineering, and Mathematics Ethical Review Committee (ERN_16-0281AP11A).

### Participants

#### Sample size and effect size calculations

*A priori* power analysis using G*Power (Faul et al., 2007) indicated that a sample size of 24 participants in each group would be required to detect a large effect size (Cohen’s *d* = 0.73) with 80% power for one-tailed between-group comparison (α = 0.05). As a reference to a prior study on reasoning in autism (Brosnan et al., 2017) which recruited 26 participants for autism and 22 for comparison group, we decided to recruit 48 participants in total, with 24 participants in each group.

#### Sample

We recruited 48 participants, with 24 participants in the autism group (9 Female, 14 Male, 1 Non-binary/Other; *M_age_* = 37.46, *SD_age_* = 15.08) and 24 participants in the non-autism group (9 Female, 14 Male, 1 Non-binary/Other; *M_age_* = 37.83, *SD_age_* = 17.49). All participants were aged 18 years or older, located in the United Kingdom, and fluent in English (all participants in the autism group and 22 out of 24 participants in the non-autism group had English as their first language). In terms of ethnicity, most participants identified as White (81.25%; see Supplementary Appendix 1) and had completed or attended higher education (77.08%; see Supplementary Appendix 2). Participants’ socioeconomic status levels were not recorded.

The autism group was recruited through various channels, including the University of Birmingham Research Team’s research participant database, student and staff mailing lists, social media platforms and flyers distributed around the University of Birmingham. The inclusion criteria for the autism group stated a clinical autism diagnosis, which was confirmed prior to recruitment and at the start of each video call. Participants provided details about their diagnoses, including diagnosis date and the profession of the professional who made the diagnosis.

The non-autism group was recruited through mailing lists and flyers at the same university, social media platforms and Prolific (https://www.prolific.co). Each participant in this group confirmed that they never received a clinical autism diagnosis and they do not identify as neurodivergent.

The levels of autistic traits for all participants were assessed to ensure the groups were distinct. The autism group reported significantly higher levels of autistic traits compared to the non-autism group; *t*(46) = 9.72, *p* < 0.001, *d* = 2.81. All participants also provided age and gender information and completed the Matrix Reasoning Item Bank (MaRs-IB; Chierchia et al., 2019), a non-verbal cognitive assessment, to achieve matched groups based on age, gender and cognitive ability (see Table 1).

**Table 1.** Demographics of autism and comparison groups.

	Autism (*N* = 24)	Comparison (*N* = 24)	Statistical test
Gender	F: 9, M: 14, O: 1	F: 9, M: 14, O: 1	χ^2^(2) = 0.000, *p* = 1.000
Age	37.46 (15.08)	37.83 (17.49)	*t*(46) = 0.80, *p* = 0.937
NVR	63.08 (19.56)	65.19 (15.62)	*t*(46) = 0.41, *p* = 0.681
AQ	36.96 (8.99)	15.25 (6.23)	*t*(46) = 9.72, *p* < 0.001

F: Female, M: Male, O: Other/Non-binary, NVR: Non-verbal reasoning, AQ: Autism Quotient. Mean scores, and Standard Deviations in parentheses next to them, are reported for each group. Age is reported in years. NVR shows the percentage of correct responses. Independent samples *t*-tests were conducted for age, NVR and AQ scores. A chi-square test was conducted for gender.

### Materials

#### Subjective thinking style

The subjective inclination towards deliberation and intuition was measured with the REI (Epstein et al., 1996), which is a 40-item self-report questionnaire, measuring the perception of engagement and ability in rationality and experientiality. The REI features four subscales, each compromising 10 items: rational engagement, rational ability, experiential engagement and experiential ability. We combined the scores for rational engagement and rational ability to assess (1) rationality, and experiential engagement and experiential ability to assess (2) experientiality. The rationality subscale measures ‘deliberation’ (‘need for cognition’, Cacioppo & Petty, 1982) with an example statement of ‘I have a logical mind’. The experientiality subscale measures ‘intuition’ (‘faith in intuition’, Epstein et al., 1996) with an example statement of ‘I believe in trusting my hunches’. This questionnaire is scored on a five-point scale ranging from 1 = *definitely not true of myself* to 5 = *definitely true of myself*. Subscale scores ranged from a minimum of 20 to a maximum of 100. The REI has strong internal consistency (rationality, α = 0.90; experientiality, α = 0.87) and reliability (rationality, *r* = ranging between 0.86 and 0.91; experientiality, *r* = ranging between 0.87 and 0.90; Pacini & Epstein, 1999).

#### Objective reasoning performance

The objective performance of cognitive reflection and intuition inhibition was measured using the recently updated CRT (Sirota & Juanchich, 2018). This test is an expanded version of CRT (Frederick, 2005), which originally consisted of three problems with open-ended response options. The CRT has been expanded with four additional problems (Toplak et al., 2014), presented with four response options in a multiple-choice format. Each problem presents one correct option that can be reached through deliberation, and one incorrect option that can be reached through intuition, along with two more incorrect options, that are neither deliberative nor intuitive. The updated version was used because the classical CRT has been extensively published, increasing the familiarity risk. The following is an example problem from the classical CRT: ‘A bat and a ball cost $1.10 in total. The bat costs a dollar more than the ball. How much does the ball cost? ____ cents’. In the updated CRT, the following options are provided for this question: ‘10 pence’, ‘5 pence’, ‘9 pence’ and ‘1 penny’. The most common answer, and the one that comes to the mind first, is 10 cents (Frederick, 2005). However, ‘10 pence’ is the incorrect and intuitive answer, while ‘5 pence’ is the correct and deliberative answer. ‘9 pence’ and ‘1 penny’ are both incorrect, but neither deliberative nor intuitive. Participants received two scores: (1) a ‘reflectiveness’ score for each correct answer and (2) an ‘intuitiveness’ score for each incorrect and intuitive answer. Therefore, a participant could get a score between 0 and 7 for each category. The multiple-choice format with four response options was chosen following Sirota and Juanchich’s (2018) suggestion for practical and methodological reasons. The problems and response options were presented randomly. The updated CRT has strong internal consistency (α = 0.71; Sirota and Juanchich, 2018).

#### The Scenario-based Comparison Task

**Figure 2.**
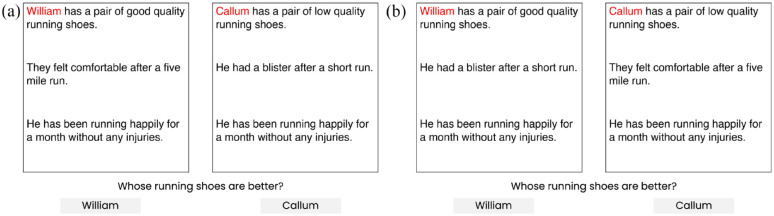
Example (a) consistent and (b) inconsistent scenario comparisons in the non-social domain. For comparisons with good outcomes, participants were asked whose [object] was better. For comparisons with bad outcomes, whose [object] was worse.

The scenario-based comparison task (adapted from Komeda et al., 2016) consisted of pairs of scenarios representing social and non-social domains. The task was designed to measure whether participants would rely on specific information when making judgements about the scenarios’ main characters. In the social domain, scenarios featured an interaction between a child and the child’s parent, while in the non-social domain, an interaction between a person and an object. Participants were asked to judge which child, in the social domain, or object, in the non-social domain, was better or worse. In this task, each scenario consisted of three lines of information: the first presented character-based information, the second presented behaviour-based information and the third presented the outcome of the scenario. Each line had either positive or negative valence. Each scenario had either a consistent or inconsistent structure, depending on whether the values of character- and behaviour-based information were aligned (both positive or both negative) or not (one positive and one negative). Consistent scenario comparisons had an expected, normatively correct answer, for example, that a ‘good’ character who behaves well is ‘better’ than a ‘bad’ character who behaves poorly. Inconsistent scenario comparisons did not have a normatively correct answer. Rather, participants’ reasoning tendencies are judged by their consistency across trials – in consistently choosing to make use of behaviour-based information or switching between using behaviour- and characteristic-based information. See Figure 1 for social and Figure 2 for non-social scenario comparisons.

The proportions of behaviour-based responses were calculated for social and non-social domains. If a participant’s response was behaviour-consistent, it was coded as 1. For instance, when asked which child was better, if a participant chose the child that was presented with positively valenced behaviour, regardless of characteristics and outcome, this was considered behaviour-consistent response. If the response was behaviour-inconsistent, it was coded as 0. For instance, when asked which child was better, if a participant chose the child that was presented with negatively valenced behaviour, regardless of characteristics and outcome, this was considered behaviour-inconsistent response. Each domain included 24 comparisons, presented twice in opposing orders of the scenarios’ main characters. Scenario main characters’ genders and positive behaviour position (left or right) were counterbalanced. Domain order (social or non-social domain first) was controlled across participants. Outcome lines’ length and word count were the same across all scenarios.

#### Autistic traits

The levels of autistic traits were measured with the Autism Spectrum Quotient (AQ; Baron-Cohen et al., 2001), which is a 50-item self-report questionnaire for adults aged 16 years and above with average or higher intelligence. Each item is presented with four options: ‘definitely agree’, ‘slightly agree’, ‘slightly disagree’ and ‘definitely disagree’. The total scores were calculated by summing participants’ responses. The total scores range from 0 to 50, with higher scores indicating higher levels of autistic traits. The AQ, widely used in clinical and non-clinical samples (Ruzich et al., 2015), demonstrates strong test–retest reliability (*r* ⩾ 0.8) and internal consistency (α ⩾ 0.7; Stevenson & Hart, 2017).

#### Non-verbal cognitive ability

Non-verbal cognitive ability was measured with the Matrix Reasoning Item Bank (MaRs-IB; Chierchia et al., 2019), using the colour-blind palette 1. The colour-blind version was selected to increase accessibility. The MaRs-IB consisted of 80 puzzles, each puzzle is a 3 × 3 grid of patterns with the pattern in the bottom-right missing. Patterns within the grid varied in shape, size, colour and position. Participants were required to select the missing pattern from four options displayed below the grid, considering the relational information based on shape, size, colour and position. Participants had 30 s to provide a response for each puzzle. In the absence of a response, the test automatically moved to the next item. The MaRs-IB had a total time limit of 8 min, but participants were not required to solve all puzzles within that time. Participants were instructed to be as fast and accurate as possible, with no penalty for incorrect answers. Puzzles were presented randomly and in a shuffle of difficulty. If a participant solved all puzzles, a reiteration of the same puzzles was presented randomly. Following Chierchia et al.’s (2019) procedure, responses to repeated puzzles were not analysed. Scores were calculated as the proportion of correct responses achieved within the 8-min time limit. The MaRs-IB has strong test–retest reliability (*r* ⩾ 0.7) and internal consistency (Kuder–Richardson 20 ⩾ 0.7; Chierchia et al., 2019).

### Procedure

Participants received an information sheet and provided consent prior to remote testing. A video call via Zoom (https://zoom.us/) was conducted to complete the scenario-based comparison task with either E.B. or Holly FitzHerbert (H.F.) (both females and non-autistic, yet H.F. has had lived experience). Participants were free to choose to keep their video on or off, with the aim of mitigating anxiety. The researcher shared screen via Zoom to the task, which was built on an online platform, Qualtrics (https://www.qualtrics.com/uk/). Each scenario comparison, with question and options, was presented on the same screen and remained on the screen until the participant verbally responded. There was no time limit during this task because time pressure was not a target. On completing this task, participants were provided with links to complete the rest of the study online on their own time. The MaRs-IB was conducted on Gorilla (https://gorilla.sc), while the REI, CRT and a demographics form were conducted on Qualtrics. Participants were compensated with £10 Amazon vouchers.

### Data analysis

IBM SPSS 29.0 was used for statistical analyses (α = 0.05). To compare whether there was a difference between groups based on subjective thinking style, a mixed-factor two-way analysis of variance (ANOVA) was conducted with within-subjects factor thinking style (rationality, experientiality) and between-subjects factor group (autism, comparison). The subscale scores of ‘rationality’ and ‘experientiality’ on the REI were used as dependent variables (DVs). To compare whether there was a difference between groups in objective reasoning performance, a Mann–Whitney U test was conducted because the scores of reflectiveness and intuitiveness were not normally distributed. The ‘reflectiveness’ and ‘intuitiveness’ scores on the CRT were used as DVs. Before moving to the main hypothesis, we conducted a mixed-factor five-way ANOVA with within-subjects factor domain type (social, non-social), consistency (consistent, inconsistent), and outcome type (good outcome, bad outcome) and between-subjects factor group (autism, comparison) and domain order (social domain first and non-social domain first) to check whether participant responses were affected by the domain order. Then, we conducted a mixed-factor three-way ANOVA with within-subjects factors domain type (social, non-social) and consistency (consistent, inconsistent), and between-subjects factor group (autism, comparison). The proportion of behaviour-based responses for scenario comparisons was used as DV.

### Community involvement

This project has benefitted from consultation with autistic people from the Birmingham Psychology Autism Research Team’s Consultancy Committee at the University of Birmingham.

## Results

### Subjective thinking style

**Figure 3.**
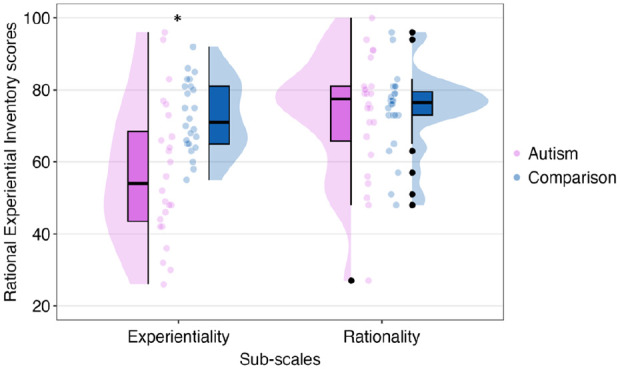
Medians and quantiles of rationality and experientiality scores by groups. Significance is shown with an asterisk. Outliers are shown with black dots.

From the REI, the mixed-factor two-way ANOVA revealed an effect of thinking style, *F*(1, 46) = 11.24, *p* = 0.002, η_*p*_^2^ = 0.20, with participants scoring higher on rationality subscale (*M* = 73.79, *SEM* = 2.04) than experientiality subscale (*M* = 64.83, *SEM* = 2.47). In addition, an effect of group was identified, *F*(1, 46) = 6.25, *p* = 0.016, η_*p*_^2^ = 0.12, with the comparison group (*M* = 73.50, *SEM* = 1.70) scoring higher than the autism group (*M* = 65.12, *SEM* = 2.88). Our hypothesis was supported by significant group × thinking style interaction, *F*(1, 46) = 7.11, *p* = 0.011, η_*p*_^2^ = 0.13. An independent-sample *t*-test revealed that the autism group (*M* = 57.08, *SEM* = 3.95) scored significantly lower on experientiality subscale than the comparison group (*M* = 72.58, *SEM* = 2.02; *t*(46) = 3.49, *p* = 0.001, *d* = 1.01, two-tailed). However, there was no significant group difference for rationality subscale, *t*(46) = 0.30, *p* = 0.764, *d* = 0.09, two-tailed (see Figure 3).

### Objective reasoning performance

**Figure 4.**
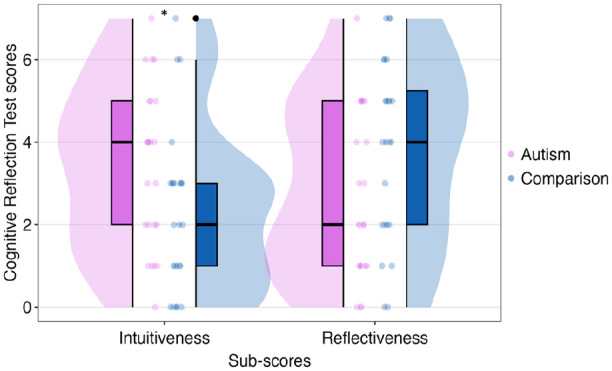
Medians and quantiles of intuitiveness and reflectiveness scores by groups. Significance is shown with an asterisk. Outliers are shown with black dots.

From the CRT, contrary to our hypothesis, the Mann–Whitney U test revealed a significant difference between groups on intuitiveness score, *U* = 189.00, *z* = 2.06, *p* = 0.039, two-tailed, with the autism group (*Mdn* = 4.00, *Mean Rank* = 28.63, *Sum of Ranks* = 687.00) scoring higher than the comparison group (*Mdn* = 2.00, *Mean Rank* = 20.38, *Sum of Ranks* = 489.00). There was also a trend towards a difference between groups on the reflectiveness score, *U* = 199.500, *z* = 1.85, *p* = 0.065 two-tailed, with the autism group (*Mdn* = 2.00, *Mean Rank* = 20.81, *Sum of Ranks* = 499.50) scoring lower than the comparison group (*Mdn* = 4.00, *Mean Rank* = 28.19, *Sum of Ranks* = 676.50) (see Figure 4).

### The Scenario-based Comparison Task

The mixed-factor five-way ANOVA revealed no significant effect of domain order (*F*(1, 46) = 0.34, *p* = 0.565, η_*p*_^2^ = 0.01). Therefore, we continued with the main analysis. The mixed-factor three-way ANOVA revealed an effect of domain type, *F*(1, 46) = 12.15, *p* = 0.001, η_*p*_^2^ = 0.21, with participants providing more behaviour-based responses for the non-social (*M* = 87.24%, *SEM* = 0.93%) than the social domain (*M* = 81.42%, *SEM* = 1.65%). There was also an effect of consistency, *F*(1, 46) = 155.77, *p* < 0.001, η_*p*_^2^ = 0.77, with participants providing more behaviour-based responses for consistent (*M* = 94.88%, *SEM* = 0.59%) than inconsistent scenario comparisons (*M* = 73.78%, *SEM* = 1.81%). However, no effect of group was identified, *F*(1, 46) = 0.04, *p* = 0.837, η_*p*_^2^ < 0.001.

In addition, there was a significant domain type × consistency interaction, *F*(1, 46) = 16.22, *p* < 0.001, η_*p*_^2^ = 0.26. To unpack this interaction, paired-sample *t*-tests were conducted. The paired-sample *t*-tests revealed significant mean differences for inconsistent scenario comparisons, *t*(47) = 3.93, *p* < 0.001, *d* = 0.57, two-tailed, non-social > social. There were no significant mean differences for consistent scenario comparisons, *t*(47) = 0.16, *p* = 0.870, *d* = 0.02, two-tailed.

**Figure 5.**
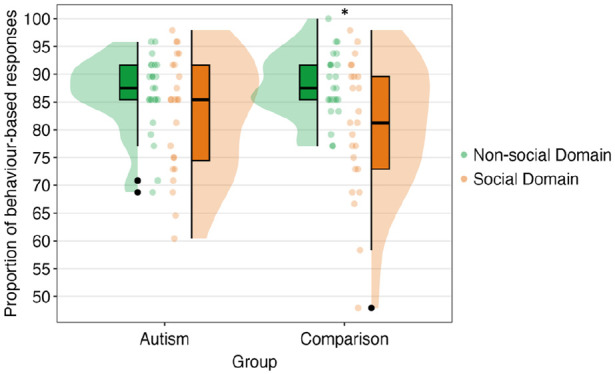
Medians and quantiles of the proportion of behaviour-based responses on the scenario-based comparison task for each group by domain. Significance is shown with an asterisk. Outliers are shown with black dots.

From the full model, no other significant interaction was identified. Neither domain type × consistency × group (*F*(1, 46) = 1.80, *p* = 0.186, η_*p*_^2^ = 0.04) nor consistency × group interaction (*F*(1, 46) = 1.65, *p* = 0.206, η_*p*_^2^ = 0.03) was significant. In addition, contrary to our main hypothesis, there was no significant group × domain type interaction, *F*(1, 46) = 1.43, *p* = 0.238, η_*p*_^2^ = 0.03. In exploratory analysis, to compare the proportion of behaviour-based responses for social and non-social domains between groups, paired-sample *t*-tests were conducted after splitting the data by group. The paired-sample *t*-tests revealed significant mean differences between social and non-social domains for the comparison, *t*(23) = 3.23, *p* = 0.004, *d* = 0.66, two-tailed, non-social > social; and not for the autism group, *t*(23) = 1.66, *p* = 0.110, *d* = 0.34, two-tailed (see Figure 5).

## Discussion

Consistent with the literature, we found that the autism group self-reported lower intuition, compared to the non-autism group. However, our findings did not support the link between autism and greater deliberation, as assessed by both objective and subjective measures of reasoning. Self-reported decreased intuition among the autism group did not align with their performance on objective measures, where the autism group scored higher in intuitiveness compared to the non-autism group. A significant difference was observed in the proportion of behaviour-based responses across the social and non-social domains among the comparison group, as opposed to the autism group. However, contrary to our main hypothesis, the interaction between domain and group did not reach to statistical significance.

### Subjective thinking style

Consistent with previous findings (e.g. Morsanyi & Hamilton, 2023; Taylor et al., 2022), autistic participants self-reported significantly lower intuition, measured by experientiality, as opposed to non-autistic participants. In addition, there was no significant difference for deliberation, measured by rationality, between groups. It is common for highly educated people to score high on rationality subscale (e.g. McLaughlin et al., 2014). This might explain the high scores on rationality for both groups. The comparison group’s rationality and experientiality scores were highly close to each other. This might suggest subjective engagement and ability in both styles or a lack of preference. In contrast, the autism group’s rationality score was significantly higher than experientiality, suggesting a perception of overreliance on a particular style. This observation could also imply a stereotype towards autistic people for being perceived as overly logical (Baron-Cohen et al., 2009). Autistic people might have been influenced by this stereotype when reporting subjective thinking style. It is crucial to be cautious when emphasising enhanced rationality in autism, as doing so might contribute to additional stereotypes. This is because it has the potential to impose further pressure on the autistic community, as these characteristics might not be representative of each autistic person.

The REI includes items that reflect real-world reasoning and decision-making. While responding to the REI, autistic people might envision situations that demand quick intuitive information processing, such as those involved in social situations (Taylor et al., 2022). Furthermore, Taylor et al. (2022) suggested that the REI might not be measuring the same components for autistic and non-autistic people, possibly due to the use of abstract and metaphorical words for some items related to intuition, such as ‘hunches’ or ‘gut feelings’.

### Objective reasoning performance

Our results from the updated CRT diverge from previous findings within the Dual Process Theory of autism which mostly used the classical version of the same measure (e.g. Brosnan et al., 2016, 2017; Lewton et al., 2019; for a review, Rozenkrantz et al., 2021). However, our results are consistent with more recent studies, such as Taylor et al. (2022) and Morsanyi and Hamilton (2023), given that the autism group did not score higher on reflectiveness than the comparison group. Specifically, the autism group provided fewer correct responses and more intuitive responses than the comparison group.

However, earlier studies had several limitations, such as small sample sizes, lack of replications, unmatched comparison groups, and absence or inconsistent assessment of cognitive ability. In addition, these studies used the classical CRT, a version that has been widely shared, contributing to the risk of increased familiarity with its items. It could be argued that reasoning studies attract people who are already interested in the field, potentially exposing them to the correct responses of this test beforehand. Furthermore, the classical version primarily consisted of numerical problems, while the updated version covers both numerical and non-numerical problems. The greater performance demonstrated by autistic people in previous studies might be attributable to their ability in numeracy, rather than to a difference in reasoning performance.

In addition, using the scenario-based comparison task, for a more direct comparison of subjective and objective decision-making of autistic people, participants could be asked to report their subjective decisions after each objective decision. This would also offer greater support for the meta-cognitive explanation of decision-making in autism (van der Plas et al., 2023), rather than the Bayesian perspective (Friston, 2016; Sevgi et al., 2020).

### Domain-specific reasoning performance

We found that participants treated the social domain differently from the non-social domain on the scenario-based comparison task. Specifically, responses in the non-social domain tended to be more behaviour-based, as opposed to the social domain. Contrary to our main hypothesis, we did not identify a significant effect of group nor did we identify an interaction between domain type and group. These findings suggested that there were no substantive differences in approaches employed by groups in their judgements.

Nonetheless, exploratory analysis did show some support for our main hypothesis. We observed a trend, hinting at a likelihood that the autism group exhibited more logical consistency across domains. In contrast, the comparison group alternated their reasoning strategies in providing significantly higher proportions of behaviour-based responses for the non-social domain, compared to the social domain. These findings were broadly in line with Komeda et al.’s (2016) work, in which the autism group displayed a higher reliance on behaviour-based information when making moral judgements. This shows some consistency with the Dual Process Theory of autism, which suggests that autistic people engage in cognitive biases less than non-autistic people and suggests these differences are particularly pronounced in social settings (e.g. Brosnan et al., 2016; De Martino et al., 2008; Shah et al., 2016). Bayesian accounts (Friston, 2016; Sevgi et al., 2020), however, would less obviously predict such results, as they suggest that cognition in autism diverges fundamentally at an information processing level. Such fundamental differences in processes *should* affect both social and non-social realms similarly, whereas here non-social reasoning was shown to be remarkably similar between autistic and non-autistic participants. Notably, any differences here are very subtle and would require larger sample sizes to detect reliability. Furthermore, any potential influence of these findings on real-world decision-making is likely to be minimal.

When we carefully match the groups based on age, gender and cognitive ability, it is plausible that previously observed group differences between autistic and non-autistic people might not manifest. The contrasting aspects of reasoning and decision-making between autistic and non-autistic people were most pronounced when autistic people were asked to report on their internal beliefs, such as confidence levels, while their actual decisions did not significantly deviate from the comparison groups (van der Plas et al., 2023). Given the incongruence between the autism group’s subjective *preference* and objective *performance* in our study, it is conceivable that this discrepancy may be due to differences in meta-cognition in autism, rather than differences in reasoning style. For instance, autistic people tend to report inaccurate levels of confidence for their correct choices (Sahuquillo-Leal et al., 2019), and their confidence levels do not correlate with their performance of error-monitoring (Doenyas et al., 2019), unlike their non-autistic counterparts who tend to report more precise confidence levels.

Furthermore, research has illuminated that greater deliberation in autism might be context-sensitive, and autistic people can be instructed to rely on intuitive reasoning (Brosnan & Ashwin, 2023b). Taken together, these observations suggest that autistic people can reason intuitively, but might encounter challenges with other aspects of decision-making, such as anxiety arising from time pressure or information overload.

### Limitations

While no significant group differences emerged in participants’ final responses on the scenario-based comparison task, our understanding of their reasoning approaches remains limited. Results obtained in an artificial environment might not necessarily reflect the complexities of real-world decision-making. The nuances of autistic people’s decision-making processes, such as time management and evaluation procedures, remain unexplored. For instance, although we did casually observe that autistic participants appeared to take longer in responding to scenario comparisons during data collection, we regrettably did not record response times. Future studies can record response times and ask for justifications for forced-choice judgements to facilitate deeper evaluation.

Our samples were reasonably well-balanced and represented a wide range of adult age groups. Specifically, the autism group included predominantly highly educated participants, who mostly identified as White, which decreases the representativeness of the results. This might be because we recruited most of our autistic participants through a research database at the University of Birmingham, where participants are enthusiastic about engaging in scientific research. However, it is important to acknowledge that unemployment and dropping out of school or not continuing to higher education are common within the autism community (Newman et al., 2011). Therefore, future studies should aim to recruit a more diverse and larger sample.

## Conclusion

We sought to test the Dual Process Theory of autism across domains by comparing the reasoning tendencies of autistic and non-autistic groups in social versus non-social domains. Contrary to the previous findings, we did not find meaningful links between autism and a tendency towards greater deliberation in performance outcomes, suggesting we should be cautious when emphasising enhanced rationality in autism. Nevertheless, consistent with existing literature, we found that the autism group, compared to the non-autism group, self-reported lower levels of intuition. Overall, our study suggests a potential disparity between subjective and objective outcomes of reasoning and decision-making among autistic participants. Future studies should aim to involve larger and more diverse samples to increase the representativeness of the results obtained.

## Supplemental Material

sj-docx-1-aut-10.1177_13623613241277055 – Supplemental material for Autistic people differ from non-autistic people subjectively, but not objectively in their reasoning Supplemental material, sj-docx-1-aut-10.1177_13623613241277055 for Autistic people differ from non-autistic people subjectively, but not objectively in their reasoning by Elif Bastan, Sarah R Beck and Andrew DR Surtees in Autism

sj-docx-2-aut-10.1177_13623613241277055 – Supplemental material for Autistic people differ from non-autistic people subjectively, but not objectively in their reasoning Supplemental material, sj-docx-2-aut-10.1177_13623613241277055 for Autistic people differ from non-autistic people subjectively, but not objectively in their reasoning by Elif Bastan, Sarah R Beck and Andrew DR Surtees in Autism

sj-docx-3-aut-10.1177_13623613241277055 – Supplemental material for Autistic people differ from non-autistic people subjectively, but not objectively in their reasoning Supplemental material, sj-docx-3-aut-10.1177_13623613241277055 for Autistic people differ from non-autistic people subjectively, but not objectively in their reasoning by Elif Bastan, Sarah R Beck and Andrew DR Surtees in Autism

sj-xlsx-4-aut-10.1177_13623613241277055 – Supplemental material for Autistic people differ from non-autistic people subjectively, but not objectively in their reasoning sj-xlsx-4-aut-10.1177_13623613241277055 for Autistic people differ from non-autistic people subjectively, but not objectively in their reasoning by Elif Bastan, Sarah R Beck and Andrew DR Surtees in Autism This article is distributed under the terms of the Creative Commons Attribution 4.0 License ( https://creativecommons.org/licenses/by/4.0/) which permits any use, reproduction and distribution of the work without further permission provided the original work is attributed as specified on the SAGE and Open Access pages ( https://us.sagepub.com/en-us/nam/open-access-at-sage).

## Notes

1. We use the terms from identity-first language (e.g. ‘autistic people’) rather than person-first language (e.g. ‘people with autism’) in keeping with the preferences of the autistic community (Gernsbacher, 2017; Keating et al., 2023; Kenny et al., 2016).
