# Supplementary material for: Autistic people differ from non-autistic people subjectively, but not objectively in their reasoning
Source: Autism. 2024 Oct 10;29(2):355–66. doi: 10.1177/13623613241277055 (PMC11816476; doi:10.1177/13623613241277055)
Supplement: sj-docx-1-aut-10.1177_13623613241277055 – Supplemental material for Autistic people differ from non-autistic people subjectively, but not objectively in their reasoning [file sj-docx-1-aut-10.1177_13623613241277055.docx]

| Olivia has a good quality jumper. |
| --- |
| It kept its shape after many washes. |
| She felt really very stylish wearing such  a smart jumper. |

| Matilda has a low quality jumper. |
| --- |
| It shrank after the very first wash. |
| She felt really very stylish wearing such  a smart jumper. |

| Olivia has a good quality jumper. |
| --- |
| It shrank after the very first wash. |
| She felt really very stylish wearing such  a smart jumper. |

| Matilda has a low quality jumper. |
| --- |
| It kept its shape after many washes. |
| She felt really very stylish wearing such  a smart jumper. |

| William has a pair of good quality  running shoes. |
| --- |
| They felt comfortable after a five mile run. |
| He has been running happily for a  month without any injuries. |

| Callum has a pair of low quality running  shoes. |
| --- |
| He had a blister after a short run. |
| He has been running happily for a  month without any injuries. |

| William has a pair of good quality  running shoes. |
| --- |
| He had a blister after a short run. |
| He has been running happily for a  month without any injuries. |

| Callum has a pair of low quality running  shoes. |
| --- |
| They felt comfortable after a five mile run. |
| He has been running happily for a  month without any injuries. |

| Isabella has a jigsaw which is well  manufactured. |
| --- |
| When she put it together, its pieces fitted each other perfectly. |
| She felt really happy putting all the  complicated puzzle pieces together. |

| Georgia has a jigsaw which is poorly  manufactured. |
| --- |
| When she put it together, its pieces didn’t fit together well. |
| She felt really happy putting all the  complicated puzzle pieces together. |

| Isabella has a jigsaw which is well  manufactured. |
| --- |
| When she put it together, its pieces didn’t fit together well. |
| She felt really happy putting all the  complicated puzzle pieces together. |

| Georgia has a jigsaw which is poorly  manufactured. |
| --- |
| When she put it together, its pieces fitted each other perfectly. |
| She felt really happy putting all the  complicated puzzle pieces together. |

| Dylan has a well written novel. |
| --- |
| It improved his language skills and kept him engaged. |
| He was happy that he found free time to  read more. |

| Adam has a poorly written novel. |
| --- |
| It wasted his time and he got bored easily. |
| He was happy that he found free time to  read more. |

| Dylan has a well written novel. |
| --- |
| It wasted his time and he got bored easily. |
| He was happy that he found free time to  read more. |

| Adam has a poorly written novel. |
| --- |
| It improved his language skills and kept him engaged. |
| He was happy that he found free time to  read more. |

| Grace has a beautiful painting in her  home. |
| --- |
| When her friends came round, she was happy they complimented the picture. |
| She felt joyful because this painting  changed the ambience in her home. |

| Alice has an ugly painting in her home. |
| --- |
| When her friends came round, she was disappointed they criticised the picture. |
| She felt joyful because this painting  changed the ambience in her home. |

| Grace has a beautiful painting in her  home. |
| --- |
| When her friends came round, she was disappointed they criticised the picture. |
| She felt joyful because this painting  changed the ambience in her home. |

| Alice has an ugly painting in her home. |
| --- |
| When her friends came round, she was happy they complimented the picture. |
| She felt joyful because this painting  changed the ambience in her home. |

| Matthew tried a tasteful coffee brand. |
| --- |
| It helped wake him up before an important meeting. |
| He was delighted to try different brands  in the new coffee shop. |

| Lewis tried a cheap coffee brand. |
| --- |
| It left him feeling sleepy before an important meeting. |
| He was delighted to try different brands  in the new coffee shop. |

| Matthew tried a tasteful coffee brand. |
| --- |
| It left him feeling sleepy before an important meeting. |
| He was delighted to try different brands  in the new coffee shop. |

| Lewis tried a cheap coffee brand. |
| --- |
| It helped wake him up before an important meeting. |
| He was delighted to try different brands  in the new coffee shop. |

| Molly has a car with a weak engine  system. |
| --- |
| It broke down on a long road trip. |
| She felt exhausted because she had to  drive all the way. |

| Poppy has a car with a powerful engine  system. |
| --- |
| It worked perfectly for a long road trip. |
| She felt exhausted because she had to  drive all the way. |

| Molly has a car with a weak engine  system. |
| --- |
| It worked perfectly for a long road trip. |
| She felt exhausted because she had to  drive all the way. |

| Poppy has a car with a powerful engine  system. |
| --- |
| It broke down on a long road trip. |
| She felt exhausted because she had to  drive all the way. |

| Alex has a cheap pen, bought from  Tesco. |
| --- |
| It was difficult to write instructions on the board with his pen. |
| He felt exhausted after writing on the  board in a lecture. |

| Charles has an expensive pen, made in  Switzerland. |
| --- |
| It was easy to write instructions on the board with his pen. |
| He felt exhausted after writing on the  board in a lecture. |

| Alex has a cheap pen, bought from  Tesco. |
| --- |
| It was easy to write instructions on the board with his pen. |
| He felt exhausted after writing on the  board in a lecture. |

| Charles has an expensive pen, made in  Switzerland. |
| --- |
| It was difficult to write instructions on the board with his pen. |
| He felt exhausted after writing on the  board in a lecture. |

| Daisy has poor quality lipstick. |
| --- |
| It faded away easily when she was at lunch with her friends. |
| She thought it was overdressing  wearing a lipstick for lunch. |

| Lily has high quality lipstick. |
| --- |
| It stayed on well when she was at lunch with her friends. |
| She thought it was overdressing  wearing a lipstick for lunch. |

| Daisy has poor quality lipstick. |
| --- |
| It stayed on well when she was at lunch with her friends. |
| She thought it was overdressing  wearing a lipstick for lunch. |

| Lily has high quality lipstick. |
| --- |
| It faded away easily when she was at lunch with her friends. |
| She thought it was overdressing  wearing a lipstick for lunch. |

| Alexander has an old cell phone. |
| --- |
| Its battery died quickly during an important call. |
| He worried about how his phone will  work during his future calls. |

| Connor has a brand-new cell phone. |
| --- |
| Its battery lasted well during an important call. |
| He worried about how his phone will  work during his future calls. |

| Alexander has an old cell phone. |
| --- |
| Its battery lasted well during an important call. |
| He worried about how his phone will  work during his future calls. |

| Connor has a brand-new cell phone. |
| --- |
| Its battery died quickly during an important call. |
| He worried about how his phone will  work during his future calls. |

| Jasmine has tough and boring slippers. |
| --- |
| It made her feel silly wearing them after work. |
| She felt exhausted after work and  slippers didn’t help her to relax. |

| Rose has soft and fluffy slippers. |
| --- |
| It made her feel comfortable wearing them after work. |
| She felt exhausted after work and  slippers didn’t help her to relax. |

| Jasmine has tough and boring slippers. |
| --- |
| It made her feel comfortable wearing them after work. |
| She felt exhausted after work and  slippers didn’t help her to relax. |

| Rose has soft and fluffy slippers. |
| --- |
| It made her feel silly wearing them after work. |
| She felt exhausted after work and  slippers didn’t help her to relax. |

| David has a weak laptop which works  slowly. |
| --- |
| An error with the laptop caused him to lose all his work. |
| He couldn’t go out as he had to  complete his work. |

| Edward has a powerful laptop which  works fast. |
| --- |
| A feature on the laptop meant that all his work was backed-up. |
| He couldn’t go out as he had to  complete his work. |

| David has a weak laptop which works  slowly. |
| --- |
| A feature on the laptop meant that all his work was backed-up. |
| He couldn’t go out as he had to  complete his work. |

| Edward has a powerful laptop which  works fast. |
| --- |
| An error with the laptop caused him to lose all his work. |
| He couldn’t go out as he had to  complete his work. |

| Lottie has a computer which is quick at  responding. |
| --- |
| It was helpful when she needed to reply to lots of emails. |
| She was very grateful that she could do  work on her computer. |

| Zoe has a computer which is slow at  responding. |
| --- |
| It was annoying as it needed to install lots of new updates. |
| She was very grateful that she could do  work on her computer. |

| Lottie has a computer which is quick at  responding. |
| --- |
| It was annoying as it needed to install lots of new updates. |
| She was very grateful that she could do  work on her computer. |

| Zoe has a computer which is slow at  responding. |
| --- |
| It was helpful when she needed to reply to lots of emails. |
| She was very grateful that she could do  work on her computer. |

| Benjamin has a good quality porcelain  cup. |
| --- |
| It kept his coffee warm this morning until he finished it. |
| He was happy he had made time for  coffee before work. |

| Sebastian has a poor quality porcelain  cup. |
| --- |
| It got broken this morning when he poured his coffee in. |
| He was happy he had made time for  coffee before work. |

| Benjamin has a good quality porcelain  cup. |
| --- |
| It got broken this morning when he poured his coffee in. |
| He was happy he had made time for  coffee before work. |

| Sebastian has a poor quality porcelain  cup. |
| --- |
| It kept his coffee warm this morning until he finished it. |
| He was happy he had made time for  coffee before work. |

| Maria has a new soft toy. |
| --- |
| She played with it lots this morning without getting bored. |
| She felt excited to show her toy to her  friends. |

| Maryam has an old worn toy. |
| --- |
| She played with it briefly this morning then got bored. |
| She felt excited to show her toy to her  friends. |

| Maria has a new soft toy. |
| --- |
| She played with it briefly this morning then got bored. |
| She felt excited to show her toy to her  friends. |

| Maryam has an old worn toy. |
| --- |
| She played with it lots this morning without getting bored. |
| She felt excited to show her toy to her  friends. |

| Joey purchased strong strings for his  violin. |
| --- |
| They lasted for a long time when he was practising. |
| He felt excited to try new strings on his  violin. |

| Rory purchased weak strings for his  violin. |
| --- |
| They broke after a short time when he was practising. |
| He felt excited to try new strings on his  violin. |

| Joey purchased strong strings for his  violin. |
| --- |
| They broke after a short time when he was practising. |
| He felt excited to try new strings on his  violin. |

| Rory purchased weak strings for his  violin. |
| --- |
| They lasted for a long time when he was practising. |
| He felt excited to try new strings on his  violin. |

| Phoebe has new headphones, which  are very good at noise cancelling. |
| --- |
| She enjoyed listening with them in the gym this morning. |
| She had an energetic work out session  with her new headphones. |

| Chloe has new headphones, which are  not good at noise cancelling. |
| --- |
| She didn’t enjoy listening with them in the gym today. |
| She had an energetic work out session  with her new headphones. |

| Phoebe has new headphones, which  are very good at noise cancelling. |
| --- |
| She didn’t enjoy listening with them in the gym today. |
| She had an energetic work out session  with her new headphones. |

| Chloe has new headphones, which are  not good at noise cancelling. |
| --- |
| She enjoyed listening with them in the gym this morning. |
| She had an energetic work out session  with her new headphones. |

| Jackson bought an expensive pair of glasses after an appointment with the optician. |
| --- |
| His eyes felt really comfortable all day at work today. |
| He felt happy after completing a long  day with his glasses. |

| Aaron bought a cheap pair of glasses  that he spotted while he shopped. |
| --- |
| His eyes felt tired after an hour at work today. |
| He felt happy after completing a long  day with his glasses. |

| Jackson bought an expensive pair of glasses after an appointment with the optician. |
| --- |
| His eyes felt tired after an hour at work today. |
| He felt happy after completing a long  day with his glasses. |

| Aaron bought a cheap pair of glasses  that he spotted while he shopped. |
| --- |
| His eyes felt really comfortable all day at work today. |
| He felt happy after completing a long  day with his glasses. |

| Piyali has a very basic pillow, which is  made of polyester. |
| --- |
| It was not comfortable and disrupted her sleep last night. |
| She was afraid that she needs to  replace her pillow. |

| Rupali has a memory foam pillow,  which is made of silk. |
| --- |
| It helped her to have a better sleep last night. |
| She was afraid that she needs to  replace her pillow. |

| Piyali has a very basic pillow, which is  made of polyester. |
| --- |
| It helped her to have a better sleep last night. |
| She was afraid that she needs to  replace her pillow. |

| Rupali has a memory foam pillow,  which is made of silk. |
| --- |
| It was not comfortable and disrupted her sleep last night. |
| She was afraid that she needs to  replace her pillow. |

| David moved to a new flat, which is in a  dodgy area. |
| --- |
| It was noisy outside, so he had a bad night’s sleep last night. |
| He felt rubbish this morning and  regretted moving into the area. |

| Elliot moved to a new flat, which is in a  safe area. |
| --- |
| It was silent outside, so he had a good night’s sleep last night. |
| He felt rubbish this morning and  regretted moving into the area. |

| David moved to a new flat, which is in a  dodgy area. |
| --- |
| It was silent outside, so he had a good night’s sleep last night. |
| He felt rubbish this morning and  regretted moving into the area. |

| Elliot moved to a new flat, which is in a  safe area. |
| --- |
| It was noisy outside, so he had a bad night’s sleep last night. |
| He felt rubbish this morning and  regretted moving into the area. |

| Elizabeth has a washing machine which  isn’t energy efficient. |
| --- |
| This morning it left a funny black mark on her favourite jumper. |
| She thought she should probably buy a  new washing machine. |

| Scarlett has a washing machine which  is energy efficient. |
| --- |
| This morning it got a nasty stain out of her favourite jumper. |
| She thought she should probably buy a  new washing machine. |

| Elizabeth has a washing machine which  isn’t energy efficient. |
| --- |
| This morning it got a nasty stain out of her favourite jumper. |
| She thought she should probably buy a  new washing machine. |

| Scarlett has a washing machine which  is energy efficient. |
| --- |
| This morning it left a funny black mark on her favourite jumper. |
| She thought she should probably buy a  new washing machine. |

| Leon has a poorly-reviewed video  game. |
| --- |
| It has low resolution graphics, so he didn’t enjoy playing it. |
| He thought playing video games is  time-consuming, and he should quit. |

| Owen has a well-reviewed video game. |
| --- |
| It has high resolution graphics, so he had fun playing it. |
| He thought playing video games is  time-consuming, and he should quit. |

| Leon has a poorly-reviewed video  game. |
| --- |
| It has high resolution graphics, so he had fun playing it. |
| He thought playing video games is  time-consuming, and he should quit. |

| Owen has a well-reviewed video game. |
| --- |
| It has low resolution graphics, so he didn’t enjoy playing it. |
| He thought playing video games is  time-consuming, and he should quit. |

| Jasmine lives in Luton, where the air  quality is generally low. |
| --- |
| This morning she got stuck in an annoying traffic jam there. |
| She decided she was getting bored of  living in the same town. |

| Catherine lives in Devon, where the  weather is clean and fresh. |
| --- |
| This morning she had a lovely walk in the park there. |
| She decided she was getting bored of  living in the same town. |

| Jasmine lives in Luton, where the air  quality is generally low. |
| --- |
| This morning she had a lovely walk in the park there. |
| She decided she was getting bored of  living in the same town. |

| Catherine lives in Devon, where the  weather is clean and fresh. |
| --- |
| This morning she got stuck in an annoying traffic jam there. |
| She decided she was getting bored of  living in the same town. |

| Michael is a regular of a noisy coffee  shop. |
| --- |
| The Wi-Fi connection there was rubbish yesterday, so he didn’t get any work done when he went there with his laptop. |
| He thought he should probably try  some new places for coffee. |

| Gabriel is a regular of a peaceful coffee  shop. |
| --- |
| The Wi-Fi connection there was good yesterday, so he got lots of work done when he went there with his laptop. |
| He thought he should probably try  some new places for coffee. |

| Michael is a regular of a noisy coffee  shop. |
| --- |
| The Wi-Fi connection there was good yesterday, so he got lots of work done when he went there with his laptop. |
| He thought he should probably try  some new places for coffee. |

| Gabriel is a regular of a peaceful coffee  shop. |
| --- |
| The Wi-Fi connection there was rubbish yesterday, so he didn’t get any work done when he went there with his laptop. |
| He thought he should probably try  some new places for coffee. |
