# Supplementary material for: Autistic people differ from non-autistic people subjectively, but not objectively in their reasoning
Source: Autism. 2024 Oct 10;29(2):355–66. doi: 10.1177/13623613241277055 (PMC11816476; doi:10.1177/13623613241277055)
Supplement: sj-docx-2-aut-10.1177_13623613241277055 – Supplemental material for Autistic people differ from non-autistic people subjectively, but not objectively in their reasoning [file sj-docx-2-aut-10.1177_13623613241277055.docx]

| George is a nice boy who likes to please his dad. |
| --- |
| He said to his dad, “Let’s go watch your favourite football team play!”, when his dad looked bored. |
| His dad smiled when he looked at his  son’s happy face. |

| James is a selfish boy who only really thinks of himself. |
| --- |
| He said to his dad, “Let’s go see my  favourite cartoon movie again!”, when his dad was busy. |
| His dad smiled when he looked at his  son’s happy face. |

| George is a nice boy who likes to  please his dad. |
| --- |
| He said to his dad, “Let’s go see my  favourite cartoon movie again!”, when  his dad was busy. |
| His dad smiled when he looked at his  son’s happy face. |

| James is a selfish boy who only really  thinks of himself. |
| --- |
| He said to his dad, “Let’s go watch your favourite football team play!”, when his dad looked bored. |
| His dad smiled when he looked at his son’s happy face. |

| Diana is a caring girl who likes to help  her dad. |
| --- |
| She wanted to clean her dad’s desk when he wasn’t there. |
| Her dad was delighted because she  found his lost watch. |

| Linda is a thoughtless girl who likes to  make a mess. |
| --- |
| She rummaged through her dad’s desk to look for something interesting. |
| Her dad was delighted because she  found his lost watch. |

| Diana is a caring girl who likes to help  her dad. |
| --- |
| She rummaged through her dad’s desk to look for something interesting. |
| Her dad was delighted because she  found his lost watch. |

| Linda is a thoughtless girl who likes to  make a mess. |
| --- |
| She wanted to clean her dad’s desk when he wasn’t there. |
| Her dad was delighted because she  found his lost watch. |

| Jack is a gentle boy who often tidies up  his stuff. |
| --- |
| He placed his toys in a box in his room. |
| His mum was really delighted with the  tidiness of the room. |

| Jacob is an aggressive boy who often  handles his toys roughly. |
| --- |
| He threw his toys in a box in the distance. |
| His mum was really delighted with the  tidiness of the room. |

| Jack is a gentle boy who often tidies up  his stuff. |
| --- |
| He threw his toys in a box in the distance. |
| His mum was really delighted with the  tidiness of the room. |

| Jacob is an aggressive boy who often  handles his toys roughly. |
| --- |
| He placed his toys in a box in his room. |
| His mum was really delighted with the  tidiness of the room. |

| Jessica is a cheerful girl who likes to  make her mum smile. |
| --- |
| She gave her mum a loving hug. |
| Her mum was glad that her daughter  could express her emotions. |

| Rebecca is a grumpy girl who gets  angry with people very quickly. |
| --- |
| She got angry and hit her mum. |
| Her mum was glad that her daughter  could express her emotions. |

| Jessica is a cheerful girl who likes to  make her mum smile. |
| --- |
| She got angry and hit her mum. |
| Her mum was glad that her daughter  could express her emotions. |

| Rebecca is a grumpy girl who gets  angry with people very quickly. |
| --- |
| She gave her mum a loving hug. |
| Her mum was glad that her daughter  could express her emotions. |

| Thomas is an easy-going boy who eats  healthy food often. |
| --- |
| He enjoyed eating the meal his mum cooked because he thought it was healthy. |
| His mum was happy to have dinner at  home with her son. |

| Joshua is a picky boy who is fussy  about food. |
| --- |
| He refused the meal his mum cooked because he had a chocolate bar before. |
| His mum was happy to have dinner at  home with her son. |

| Thomas is an easy-going boy who eats  healthy food often. |
| --- |
| He refused the meal his mum cooked because he had a chocolate bar before. |
| His mum was happy to have dinner at  home with her son. |

| Joshua is a picky boy who is fussy  about food. |
| --- |
| He enjoyed eating the meal his mum cooked because he thought it was healthy. |
| His mum was happy to have dinner at  home with her son. |

| Barbara is a hard-working girl who  performs well at school. |
| --- |
| She studied really very hard to get a good score on a test. |
| Her dad was really happy to see her  high test scores. |

| Melissa is a lazy girl who performs  poorly at school. |
| --- |
| She copied her friend’s answers to get a good score on a test. |
| Her dad was really happy to see her  high test scores. |

| Barbara is a hard-working girl who  performs well at school. |
| --- |
| She copied her friend’s answers to get a good score on a test. |
| Her dad was really happy to see her  high test scores. |

| Melissa is a lazy girl who performs  poorly at school. |
| --- |
| She studied really very hard to get a good score on a test. |
| Her dad was really happy to see her  high test scores. |

| Harry is an impatient boy who gets  frustrated with other people. |
| --- |
| He shook his dad’s beer-can vigorously before he gave it to him. |
| His dad’s clothes got wet when the beer  jumped from the can. |

| Charlie is a caring boy who likes  making his dad happy. |
| --- |
| He wanted to give his dad a can of his dad’s favourite beer. |
| His dad’s clothes got wet when the beer  jumped from the can. |

| Harry is an impatient boy who gets  frustrated with other people. |
| --- |
| He wanted to give his dad a can of his dad’s favourite beer. |
| His dad’s clothes got wet when the beer  jumped from the can. |

| Charlie is a caring boy who likes  making his dad happy. |
| --- |
| He shook his dad’s beer-can vigorously before he gave it to him. |
| His dad’s clothes got wet when the beer  jumped from the can. |

| Emily is a naughty girl who likes to do  dangerous things. |
| --- |
| She climbed on the table to reach sweets on a shelf and knocked over a vase. |
| Her mum was sad because her  favourite vase got badly broken. |

| Betty is a considerate girl who likes to  help her mum. |
| --- |
| She cleaned up the table to try to help her mum and knocked over a vase. |
| Her mum was sad because her  favourite vase got badly broken. |

| Emily is a naughty girl who likes to do  dangerous things. |
| --- |
| She cleaned up the table to try to help her mum and knocked over a vase. |
| Her mum was sad because her  favourite vase got badly broken. |

| Betty is a considerate girl who likes to  help her mum. |
| --- |
| She climbed on the table to reach sweets on a shelf and knocked over a vase. |
| Her mum was sad because her  favourite vase got badly broken. |

| Peter is a careless boy who often  damages his parents’ things. |
| --- |
| He dropped his mum’s vase when he was climbing on the table. |
| His mum sadly tidied up the broken  vase into the bin. |

| Oliver is an active boy who likes to help  with housework. |
| --- |
| He dropped his mum’s vase when he was trying to wipe it. |
| His mum sadly tidied up the broken  vase into the bin. |

| Peter is a careless boy who often  damages his parents’ things. |
| --- |
| He dropped his mum’s vase when he was trying to wipe it. |
| His mum sadly tidied up the broken  vase into the bin. |

| Oliver is an active boy who likes to help  with housework. |
| --- |
| He dropped his mum’s vase when he was climbing on the table. |
| His mum sadly tidied up the broken  vase into the bin. |

| Karen is a naughty girl who likes  making mischief. |
| --- |
| She made her mum a meal with tomatoes she knew were rotten. |
| Her mum had a stomach-ache because  the tomatoes were terrible. |

| Helen is a kind girl who likes to cook. |
| --- |
| She gave her mum pasta which she made with juicy ripe tomatoes. |
| Her mum had a stomach-ache because  the tomatoes were terrible. |

| Karen is a naughty girl who likes  making mischief. |
| --- |
| She gave her mum pasta which she made with juicy ripe tomatoes. |
| Her mum had a stomach-ache because  the tomatoes were terrible. |

| Helen is a kind girl who likes to cook. |
| --- |
| She made her mum a meal with tomatoes she knew were rotten. |
| Her mum had a stomach-ache because  the tomatoes were terrible. |

| Albert is an aggressive boy who likes  doing dangerous things. |
| --- |
| He was with his dad on bonfire night and pointed a firework at him to scare him. |
| His dad had a really severe burn from  the firework. |

| Robert is a grateful boy who cares  about his family. |
| --- |
| He was with his dad on bonfire night and brought him a lit firework to help him. |
| His dad had a really severe burn from  the firework. |

| Albert is an aggressive boy who likes  doing dangerous things. |
| --- |
| He was with his dad on bonfire night and brought him a lit firework to help him. |
| His dad had a really severe burn from  the firework. |

| Robert is a grateful boy who cares  about his family. |
| --- |
| He was with his dad on bonfire night and pointed a firework at him to scare him. |
| His dad had a really severe burn from  the firework. |

| Emma is an annoying girl who likes to  play jokes on other people. |
| --- |
| She put a lot of salt in her dad’s coffee to see her dad’s reaction. |
| Her dad frowned after drinking the  coffee, which was disgusting. |

| Lisa is a generous girl who likes to do  nice things for people. |
| --- |
| She put a lot of sugar in her dad’s coffee as a treat for him. |
| Her dad frowned after drinking the  coffee, which was disgusting. |

| Emma is an annoying girl who likes to  play jokes on other people. |
| --- |
| She put a lot of sugar in her dad’s coffee as a treat for him. |
| Her dad frowned after drinking the  coffee, which was disgusting. |

| Lisa is a generous girl who likes to do  nice things for people. |
| --- |
| She put a lot of salt in her dad’s coffee to see her dad’s reaction. |
| Her dad frowned after drinking the  coffee, which was disgusting. |

| Mason is an energetic boy who is good  at most sports. |
| --- |
| He played baseball in the park with his dad, who likes baseball lots. |
| His dad was very happy to play  baseball with his son. |

| Logan is a naughty boy who often fights  with his mum. |
| --- |
| He played baseball with his dad because he hates being with his mum. |
| His dad was very happy to play  baseball with his son. |

| Mason is an energetic boy who is good  at most sports. |
| --- |
| He played baseball with his dad because he hates being with his mum. |
| His dad was very happy to play  baseball with his son. |

| Logan is a naughty boy who often fights  with his mum. |
| --- |
| He played baseball in the park with his dad, who likes baseball lots. |
| His dad was very happy to play  baseball with his son. |

| Julie is an ambitious girl who works hard  on various things. |
| --- |
| She gave her mum a cookie she’d made  with delicious chocolate. |
| Her mum ate a bite and said that it was absolutely delicious. |

| Olive is a lazy girl who doesn’t like to  work hard. |
| --- |
| She gave her mum a cookie she’d made  with rancid butter. |
| Her mum ate a bite and said that it was absolutely delicious. |

| Julie is an ambitious girl who works hard  on various things. |
| --- |
| She gave her mum a cookie she’d made  with rancid butter. |
| Her mum ate a bite and said that it was absolutely delicious. |

| Olive is a lazy girl who doesn’t like to  work hard. |
| --- |
| She gave her mum a cookie she’d made  with delicious chocolate. |
| Her mum ate a bite and said that it was absolutely delicious. |

| Riley is a confident boy who really likes  making things. |
| --- |
| He made a plastic model of a car without the help of his dad. |
| His dad was happy with the completed  plastic model of the car. |

| Daniel is a nervous boy who is over-  reliant on others. |
| --- |
| He complained until his dad made a plastic model of a car with him. |
| His dad was happy with the completed  plastic model of the car. |

| Riley is a confident boy who really likes  making things. |
| --- |
| He complained until his dad made a plastic model of a car with him. |
| His dad was happy with the completed  plastic model of the car. |

| Daniel is a nervous boy who is over-  reliant on others. |
| --- |
| He made a plastic model of a car without the help of his dad. |
| His dad was happy with the completed  plastic model of the car. |

| Amber is a generous girl who likes to  share her food. |
| --- |
| She shared some of her ice cream with her mum because she thought it was really delicious. |
| Her mum very much enjoyed eating  some refreshing ice cream. |

| Anabelle is a picky girl who only eats  unhealthy junk food. |
| --- |
| She tried a little ice cream and thought it was terrible, so gave it to her mum. |
| Her mum very much enjoyed eating  some refreshing ice cream. |

| Amber is a generous girl who likes to  share her food. |
| --- |
| She tried a little ice cream and thought it was terrible, so gave it to her mum. |
| Her mum very much enjoyed eating  some refreshing ice cream. |

| Anabelle is a picky girl who only eats  unhealthy junk food. |
| --- |
| She shared some of her ice cream with her mum because she thought it was really delicious. |
| Her mum very much enjoyed eating  some refreshing ice cream. |

| Noah is a lively boy who often helps his  dad. |
| --- |
| When he was fishing with his dad, he tried to help his dad by grabbing a fish he had hooked. |
| His dad was happy that his son was  helping him with fishing. |

| Liam is an impatient boy who does  things without thinking. |
| --- |
| When he was fishing with his dad, he tried to grab a fish that his dad hooked to annoy him. |
| His dad was happy that his son was  helping him with fishing. |

| Noah is a lively boy who often helps his  dad. |
| --- |
| When he was fishing with his dad, he tried to grab a fish that his dad hooked to annoy him. |
| His dad was happy that his son was  helping him with fishing. |

| Liam is an impatient boy who does  things without thinking. |
| --- |
| When he was fishing with his dad, he tried to help his dad by grabbing a fish he had hooked. |
| His dad was happy that his son was  helping him with fishing. |

| Nancy is a considerate girl who helps  with gardening at home. |
| --- |
| She gave her dad a delicious looking cucumber she had grown by herself. |
| Her dad carefully wiped and ate the  fresh cucumber happily. |

| Cindy is an annoying girl who likes to  irritate other people. |
| --- |
| She gave her dad a cucumber to eat that was covered in mud. |
| Her dad carefully wiped and ate the  fresh cucumber happily. |

| Nancy is a considerate girl who helps  with gardening at home. |
| --- |
| She gave her dad a cucumber to eat that was covered in mud. |
| Her dad carefully wiped and ate the  fresh cucumber happily. |

| Cindy is an annoying girl who likes to  irritate other people. |
| --- |
| She gave her dad a delicious looking cucumber she had grown by herself. |
| Her dad carefully wiped and ate the  fresh cucumber happily. |

| Max is a mean boy who likes to tease  animals. |
| --- |
| He wanted to surprise his dad and gave him an untrained dog. |
| His dad’s hand was bitten by the  disturbed dog and bled. |

| Lucas is a kind boy who likes lots of  animals. |
| --- |
| He gave his dad a dog because he thought it was cute. |
| His dad’s hand was bitten by the  disturbed dog and bled. |

| Max is a mean boy who likes to tease  animals. |
| --- |
| He gave his dad a dog because he thought it was cute. |
| His dad’s hand was bitten by the  disturbed dog and bled. |

| Lucas is a kind boy who likes lots of  animals. |
| --- |
| He wanted to surprise his dad and gave him an untrained dog. |
| His dad’s hand was bitten by the  disturbed dog and bled. |

| Laura is a bullying girl who likes to  make fun of people. |
| --- |
| She told her friends about her mum’s recent failure at work. |
| Her mum was embarrassed because  there were rumours among the parents. |

| Amelia is a cheerful girl who likes to talk  to other people. |
| --- |
| She told her friends about her mum’s recent success at work. |
| Her mum was embarrassed because  there were rumours among the parents. |

| Laura is a bullying girl who likes to  make fun of people. |
| --- |
| She told her friends about her mum’s recent success at work. |
| Her mum was embarrassed because  there were rumours among the parents. |

| Amelia is a cheerful girl who likes to talk  to other people. |
| --- |
| She told her friends about her mum’s recent failure at work. |
| Her mum was embarrassed because  there were rumours among the parents. |

| Freddie is a selfish boy who is very  demanding. |
| --- |
| He demanded his mum to take him to the pool when his mum was very tired. |
| His mum caught a cold because the  pool was cold. |

| Archie is an active boy who likes to  exercise. |
| --- |
| He went to the pool with his mum to show her how well he could swim. |
| His mum caught a cold because the  pool was cold. |

| Freddie is a selfish boy who is very  demanding. |
| --- |
| He went to the pool with his mum to show her how well he could swim. |
| His mum caught a cold because the  pool was cold. |

| Archie is an active boy who likes to  exercise. |
| --- |
| He demanded his mum to take him to the pool when his mum was very tired. |
| His mum caught a cold because the  pool was cold. |

| Amy is an impulsive girl who likes  making mischief. |
| --- |
| She purposely flipped her dad’s one- thousand-piece jigsaw puzzle over and broke it apart. |
| Her dad got upset because he couldn’t  continue the jigsaw puzzle. |

| Mary is a bright girl who has varied  interests. |
| --- |
| She wanted to help her dad with his jigsaw puzzle but accidentally flipped it over. |
| Her dad got upset because he couldn’t  continue the jigsaw puzzle. |

| Amy is an impulsive girl who likes  making mischief. |
| --- |
| She wanted to help her dad with his jigsaw puzzle but accidentally flipped it over. |
| Her dad got upset because he couldn’t  continue the jigsaw puzzle. |

| Mary is a bright girl who has varied  interests. |
| --- |
| She purposely flipped her dad’s one- thousand-piece jigsaw puzzle over and broke it apart. |
| Her dad got upset because he couldn’t  continue the jigsaw puzzle. |

| Alfie is a careless boy who does lots of  bad things. |
| --- |
| He was playing with his mum’s umbrella, and it got caught in a tree. |
| His mum couldn't go out on a rainy day  without her umbrella. |

| Henry is a helpful boy who does things  for other people. |
| --- |
| He was trying to fix his mum’s umbrella, but it got broken much worse. |
| His mum couldn't go out on a rainy day  without her umbrella. |

| Alfie is a careless boy who does lots of  bad things. |
| --- |
| He was trying to fix his mum’s umbrella, but it got broken much worse. |
| His mum couldn't go out on a rainy day  without her umbrella. |

| Henry is a helpful boy who does things  for other people. |
| --- |
| He was playing with his mum’s umbrella, and it got caught in a tree. |
| His mum couldn't go out on a rainy day  without her umbrella. |

| Ella is a spoilt girl who neglects her  parents. |
| --- |
| She forgot her mum’s birthday and gave her an unwanted present of her own. |
| Her mum was sad because of aging. |

| Anna is a grateful girl who appreciates  her parents. |
| --- |
| She made a thoughtful gift for her mum’s birthday and gave it to her. |
| Her mum was sad because of aging. |

| Ella is a spoilt girl who neglects her  parents. |
| --- |
| She made a thoughtful gift for her mum’s birthday and gave it to her. |
| Her mum was sad because of aging. |

| Anna is a grateful girl who appreciates  her parents. |
| --- |
| She forgot her mum’s birthday and gave her an unwanted present of her own. |
| Her mum was sad because of aging. |
