# Supplementary material for: Autistic people differ from non-autistic people subjectively, but not objectively in their reasoning
Source: Autism. 2024 Oct 10;29(2):355–66. doi: 10.1177/13623613241277055 (PMC11816476; doi:10.1177/13623613241277055)
Supplement: sj-docx-3-aut-10.1177_13623613241277055 – Supplemental material for Autistic people differ from non-autistic people subjectively, but not objectively in their reasoning [file sj-docx-3-aut-10.1177_13623613241277055.docx]

**Appendices**

| **Appendix 1** | | |
| --- | --- | --- |
| *Participants’ ethnicity information* | | |
| **Ethnic group** | **Autism**  **(*N* = 24)** | **Control**  **(*N* = 24)** |
| White—English/Welsh/Scottish/Northern Irish/British | 20 | 17 |
| White—Other | 0 | 2 |
| Mixed/Multiple Ethnics Groups—White and Black Caribbean | 1 | 0 |
| Mixed/Multiple Ethnics Groups—White and Black African | 0 | 2 |
| Mixed/Multiple Ethnics Groups—White and Asian | 1 | 0 |
| Asian/Asian British—Indian | 1 | 0 |
| Asian/Asian British—Chinese | 0 | 1 |
| Asian/Asian British—Other (Tamil) | 0 | 1 |
| Black/African/Caribbean/Black British—Caribbean | 1 | 0 |
| Prefer not to say | 0 | 1 |
| Total | 24 | 24 |

| **Appendix 2** | | |
| --- | --- | --- |
| *Participants’ education levels* | | |
| **Education level** | **Autism**  **(*N* = 24)** | **Control**  **(*N* = 24)** |
| Graduate or professional degree (MA, MS, MBA, PhD, JD, MD, DDS) | 5 | 9 |
| University bachelor’s degree | 8 | 8 |
| Some university but no degree | 4 | 3 |
| Vocational or similar | 2 | 0 |
| Completed secondary school | 4 | 4 |
| Some secondary | 1 | 0 |
| Total | 24 | 24 |
